# The impact of the *PNPLA3* rs738406 C>G polymorphism on hepatocellular carcinoma risk in Brazilian patients with chronic hepatitis C and advanced fibrosis

**DOI:** 10.1016/j.clinsp.2025.100797

**Published:** 2025-10-14

**Authors:** Claudia Maccali, Isabel Veloso Alves Pereira, Lisa Rodrigues da Cunha Saud, Regiane Saraiva de Souza Melo Alencar, Arthur Ivan Nobre Oliveira, Jose Tadeu Stefano, Michele Soares Gomes Gouvea, Joyce Matie Kinoshita da Silva Etto, Paulo Herman, João Renato Rebello Pinho, Rafael Soares Nunes Pinheiro, Wellington Andraus, Luiz Augusto Carneiro D’Albuquerque, Mario Guimarães Pessoa, Aline Lopes Chagas, Claudia Pinto Marques Souza de Oliveira

**Affiliations:** a Laboratório de Gastroenterologia Clínica e Experimental LIM-07, Division of Clinical Gastroenterology and Hepatology, Department of Gastroenterology, Hospital das Clínicas, Faculdade de Medicina, Universidade de São Paulo (HCFMUSP), São Paulo, SP, Brazil; b Division of Hepatology, Department of Gastroenterology, Instituto do Câncer do Estado de São Paulo, Hospital das Clínicas, Faculdade de Medicina, Universidade de São Paulo (HCFMUSP), São Paulo, SP, Brazil; c Division of Clinical Gastroenterology and Hepatology, Department of Gastroenterology, Hospital das Clínicas, Faculdade de Medicina, Universidade de São Paulo (HCFMUSP), São Paulo, SP, Brazil; d Laboratório de Gastroenterologia e Hepatologia Tropical, Instituto de Medicina Tropical, Departamento de Gastroenterologia, Faculdade de Medicina da Universidade de São Paulo (FMUSP), São Paulo, SP, Brazil; e Liver Surgery Unit, Department of Gastroenterology, Hospital das Clínicas, Faculdade de Medicina, Universidade de São Paulo (HCFMUSP), São Paulo, SP, Brazil; f Liver and Abdominal Transplantation, Department of Gastroenterology, Hospital das Clínicas, Faculdade de Medicina, Universidade de São Paulo (HCFMUSP), São Paulo, SP, Brazil

**Keywords:** Chronic hepatitis C, Hepatocellular carcinoma, *PNPLA3*, Biomarkers, Risk stratification

## Abstract

**Introduction:**

The *PNPLA3* rs738409 C>G polymorphism has been associated with fibrosis and steatosis in Chronic Hepatitis C (CHC) patients. However, its impact on Hepatocellular Carcinoma (HCC) development in this population remains unclear. This study aimed to evaluate the impact of the PNPLA3 polymorphism on the risk of HCC development in CHC patients in Brazil.

**Methods:**

This retrospective case-control study included 235 CHC patients with advanced fibrosis, 119 with HCC, and 116 without HCC. The PNPLA3 polymorphism was analyzed using both the dominant and recessive models. Multivariate logistic regression was performed to assess factors independently associated with HCC development.

**Results:**

The HCC group exhibited a higher proportion of male patients (p < 0.0001), alcohol abuse (p < 0.001), tobacco consumption (p < 0.0001), and ALBI grade 2‒3 (p < 0.001). The PNPLA3 genotype frequencies were CC 45.4%, CG 46.2% and GG 8.4% in the HCC group, and CC 51.7%, CG 40.5% and GG 7.8% in the non-HCC group. No significant differences in genotype frequencies were observed for the dominant model (p = 0.33) or the recessive model (p = 0.92). Gender (p = 0.001), tobacco consumption (p < 0.001), and ALBI score (p = 0.012) were identified as independent factors associated with HCC development. However, the *PNPLA3* G allele was not an independent factor associated with HCC risk in either the univariate analysis (OR = 1.15, 95% CI 0.63‒2.08, p = 0.65) or the multivariate analysis (OR = 0.99, 95% CI 0.49‒2.00, p = 0.98).

**Conclusion:**

The *PNPLA3* rs738409 polymorphism was not associated with HCC development in CHC patients with advanced fibrosis in the Brazilian population. Further studies are required to confirm this finding.

## Highlights

• *PNPLA3* rs738409 polymorphism was associated with fibrosis and steatosis in CHC patients.
• The impact on HCC risk is not yet well defined.
• This was the first evaluation of this association in the Brazilian population.
• *PNPLA3* rs738409 polymorphism was not associated with HCC risk in the studied population.

## Introduction

Chronic Hepatitis C (CHC) is a major cause of Hepatocellular Carcinoma (HCC) worldwide.^1^ To date, CHC remains the leading cause of cirrhosis and HCC in Brazil.^2^ The most important factor associated with HCC development in CHC patients is the presence of cirrhosis, with an annual incidence of 1 %‒6 %.^1^ Furthermore, the Hepatitis C Virus (HCV) can induce hepatocarcinogenesis by downregulating tumor-suppressor genes, activating oncogenes, modulating the immune response, and causing epigenetic alterations that promote HCC development.^3^ Additionally, patients may present concomitant risk factors that increase the risk of HCC, such as type 2 diabetes mellitus (T2DM), alcohol and tobacco consumption, and genetic predisposition.^4^

With the improvement in Sustained Virologic Response (SVR) rates following treatment with Direct-Acting Antiviral Agents (DAAs), there has been a significant reduction in the risk of HCC after achieving SVR.^5^ However, some factors persist post-SVR, such as comorbidities, genetic predisposition, and often the degree of fibrosis.^4^ As a result, many patients remain at high risk of developing HCC even after achieving SVR, particularly those with cirrhosis prior to treatment.^4^ Moreover, patients with lower-grade fibrosis may also present additional risk factors, increasing their risk of developing HCC.^3^

Despite these advances, HCC risk stratification in CHC patients remains a challenge.^3^ Currently, the European Association for the Study of the Liver (EASL) and the American Association for the Study of Liver Diseases (AASLD) guidelines recommend screening based solely on the degree of fibrosis.^6^^,^^7^ Therefore, there is an unmet need for new biomarkers to better stratify the risk of HCC.^3^

With the advent of Genome-Wide Association Studies (GWAS), several Single-Nucleotide Polymorphisms (SNPs) have been identified as associated with the development of various diseases.^8^ The Patatin-like Phospholipase domain containing 3 (*PNPLA3*) gene plays a key role in lipid droplet remodeling and Very Low-Density Lipoprotein (VLDL) metabolism, and the rs738409 *C* > *G* polymorphism has been linked to liver diseases, including Steatotic Liver Disease (SLD).^9^ The G allele may influence disease severity and the development of HCC in this population.^10^^,^^11^ In CHC, the *PNPLA3* recessive genotype has been independently associated with fibrosis degree and steatosis, although this association using the dominant model remains controversial.^9^^,^^12^ Furthermore, the impact of this polymorphism on HCC development is not yet well defined, regardless of the model.^9^^,^^13^ In this study, the authors aimed to evaluate the influence of the *PNPLA3* rs738409 *C* > *G* polymorphism on HCC risk in CHC patients with advanced fibrosis in the Brazilian population.

## Materials and methods

### Study design and patient selection

This single-center case-control study compared two cohorts of patients with CHC and advanced fibrosis, one cohort with HCC and another without.

The inclusion criteria for the HCC group were a previous history of CHC and a diagnosis of HCC according to EASL or AASLD guidelines.^6^^,^^7^ The exclusion criteria for this group included coinfection with Hepatitis B Virus (HBV) or Human Immunodeficiency Virus (HIV), the presence of other chronic liver diseases, absence of clinical data in medical records, and the presence of other tumors or absence of HCC in the surgical specimen or explant.

For the non-HCC group, the inclusion criteria were age ≥ 18-years, confirmed CHC with histopathological diagnosis, and advanced fibrosis (Metavir F3 and F4). The exclusion criteria included a diagnosis of HCC, other chronic liver diseases, coinfection with HIV or HBV, prior liver transplantation, and absence of clinical data in medical records.

The non-HCC group included 116 patients with CHC and advanced fibrosis who attended the outpatient units of the Departments of Gastroenterology and Infectious Diseases of the HCFMUSP between 2014 and 2016, with a median follow-up time of 7.06 ± 4.15 years. The HCC group included 119 patients with HCV-related HCC, consisting of 89 patients who underwent liver transplantation or resection between 2013 and 2019 at the Hospital das Clínicas da Faculdade de Medicina da Universidade de São Paulo (HC-FMUSP) and 30 patients from the outpatient units who developed HCC. Additionally, 134 healthy controls were included to analyze the frequencies of the *PNPLA3* rs738409 *C* > *G* polymorphism genotypes.

### Variables evaluated

Demographic, anthropometric, and clinical data from all patients included age, sex, race, HCV genotype, HCV treatment, presence of cirrhosis, portal hypertension, T2DM, Systemic Arterial Hypertension (SAH), history of alcohol and tobacco consumption, and Body Mass Index (BMI). For the non-HCC group, the last follow-up was defined as the most recent outpatient visit or imaging exam for HCC screening.

Laboratory tests were performed on the day of liver biopsy for the non-HCC group and at HCC diagnosis for the HCC group. Serum biochemistry analyzed in both groups included albumin, total bilirubin, platelets, serum glucose, Prothrombin Time/International Normalization Ratio (PT/INR), Alpha-Fetoprotein (AFP), alanine Aminotransferase (ALT), Aspartate Aminotransferase (AST) Gamma-Glutamyl Transferase (GGT), creatinine, total cholesterol, High-Density Lipoprotein (HDL), Low-Density Lipoprotein (LDL), and triglycerides. Some scores were calculated, including the Child-Pugh score, Model of End-stage Liver Disease (MELD), and Albumin-Bilirubin (ALBI) score.

The presence of steatosis was evaluated in the liver tissue using surgical specimens, explants, or liver biopsies from the HCC group, and liver biopsies for the non-HCC group.

Based on previous studies, the *PNPLA3* was analyzed using primarily the dominant model (CC vs CG/GG).^14^^,^^15^ In addition, the recessive model (GG vs. CC/CG) was also applied.

### DNA extraction and *PNPLA3* rs738409 genotyping

DNA was isolated from 200 μL of blood using the QIAamp DNA Blood Mini Kit (QIAGEN, Hilden, Germany). The *PNPLA3* rs738409 *C* > *G* was genotyped using TaqMan assay for allelic discrimination (Applied Biosystems, Thermo Fisher Brand, Foster City, CA, USA) according to the manufacturer's instructions. Direct genotyping was performed on random samples to validate the results, and quality control was carried out to ensure reproducibility.

### Ethical considerations

This study was conducted in accordance with the STROBE (Strengthening the Reporting of Observational Studies in Epidemiology) guidelines and was approved by the Ethics Committee of the Hospital das Clínicas da Faculdade de Medicina da Universidade de São Paulo (CAAE 16,828,313.0.0000.0068 and CAPPESQ 294,198). The protocol followed the Declaration of Helsinki (revised in Fortaleza, Brazil, October 2013). Informed consent was obtained from all participants.

### Statistical analysis

Data were analyzed using JAMOVI® software version 2.5. Qualitative variables were expressed as percentages and analyzed using the Chi-Square and Kruskal-Wallis tests. Quantitative variables were described as mean ± standard deviation and analyzed using Student's *t*-test and ANOVA, followed by the Tukey post hoc test for multiple comparisons. The homogeneity of variances was assessed using Levene's test. The predictive logistic regression model was evaluated using the Cox-Snell test.

## Results

### Patient characteristics

A total of 235 patients were enrolled in this study. The characteristics described below are summarized in Table 1. The mean age was higher in the HCC group (59.4 years vs. 56.3 years, *p* = 0.03), and most individuals were Caucasian in both groups, with no significant statistical difference. In the HCC group, there was a higher proportion of males and a lower proportion of females (74.8 % vs. 37.1 % and 25.2 % vs. 62.9 %, *p* < 0.0001, respectively). History of alcohol dependence (44.9 % vs. 12.2 %, *p* < 0.001) and tobacco consumption (67.24 % vs. 23 %, *p* < 0.0001) were more frequent in the HCC group. However, SAH, obesity, and steatosis were less frequent in the HCC group (38.65 % vs. 57.5 %, *p* < 0.0001; 20.8 % vs. 32.7 %, *p* = 0.043; 48.49 % vs. 64.35 %, *p* = 0.02, respectively). There was also a significant difference in the proportions of HCV genotypes, with a lower proportion of genotype 1 and a higher proportion of genotype 3 in the HCC group compared to the non-HCC group (65.86 vs. 75 % and 34.15 % vs. 20.7 %, *p* = 0.04). No significant differences were found regarding the presence of T2DM.

**Table 1:** Patient’s clinical and demographic characteristics.

**Variables** (*n* = non-HCC / HCC)		**Groups**		**p**
		**Non-HCC**	**HCC**	
**Age (years)** (*n* = 116 / 119)	Mean ± SD	56.35 ± 12.21	59.4 ± 8.52	0.03*
**Gender n (%)** (*n* = 116 / 119)	Female	73 (62.9 %)	30 (25.2 %)	<0.0001*
	Male	43 (37.1 %)	89 (74.8 %)	
**Ethnicity** (*n* = 116 / 118)	White	90 (77.59 %)	95 (80.50)	0.96
	Black	10 (8.62 %)	9 (7.64 %)	
	Mixed	15 (12.93 %)	13 (11.01 %)	
	Yellow	1 (0.86 %)	1 (0.85 %)	
**Alcohol** (*n* = 115 / 116)	Yes	14 (12.2 %)	45 (38.8 %)	<0.001*
	No	101 (87.8 %)	71 (61.2 %)	
**Tobacco** (*n* = 113 / 116)	Yes	26 (23.0 %)	78 (67.24 %)	<0.0001*
	No	87 (77.0 %)	38 (32.76 %)	
**T2DM** (*n* = 113 / 119)	Yes	33 (29.2 %)	34 (28.6 %)	0.92
	No	80 (70.8 %)	85 (71.4 %)	
**SAH** (*n* = 113 / 119)	Yes	65 (57.5 %)	46 (38.65 %)	0.004*
	No	48 (42.5 %)	73 (61.35 %)	
**Liver steatosis** (*n* = 115 / 99)	Yes	74 (64.35 %)	48 (48.49 %)	0.02*
	No	41 (35.65 %)	51 (51.51 %)	
**Obesity** (*n* = 113 / 115)	Non-obese	76 (67.3 %)	91 (79.2 %)	0.043*
	Obese	37 (32.7 %)	24 (20.8 %)	
**ALBI** (*n* = 88 / 111)	1	58 (65.90 %)	47 (42.34 %)	<0.001*
	2‒3	30 (34.10 %)	64 (57.66 %)	
**BMI (Kg/m^2^)** (*n* = 113 / 115)	Mean ± SD	27.85 ± 5.05	26.79 ± 4.91	0.11
**HCV genotype** (*n* = 116 / 82)	1	87 (75.0 %)	54 (65.85 %)	0.04*
	2	3 (2.6 %)	0	
	3	24 (20.7 %)	28 (34.15 %)	
	4	2 (1.7 %)	0	

ALBI, Albumin-Bilirubin; BMI, Body Mass Index; HCC, Hepatocellular Carcinoma; HCV, Hepatitis C Virus; SAH, Systemic Arterial Hypertension; SD, Standard Deviation; T2DM, Type 2 Diabetes Mellitus.

Assessing only cirrhotic patients (Supplementary Table 1), there was a predominance of Child-Pugh A and MELD < 15 patients in both groups, with no statistical difference. However, the HCC group presented higher ALBI scores (ALBI 1 42.34 % vs. 65.9 % and ALBI 2–3 57.66 % vs. 34.21 %, *p* = 0.002) and more portal hypertension (77.3 % vs. 65.17 %, *p* = 0.05).

**Fig. 1:**
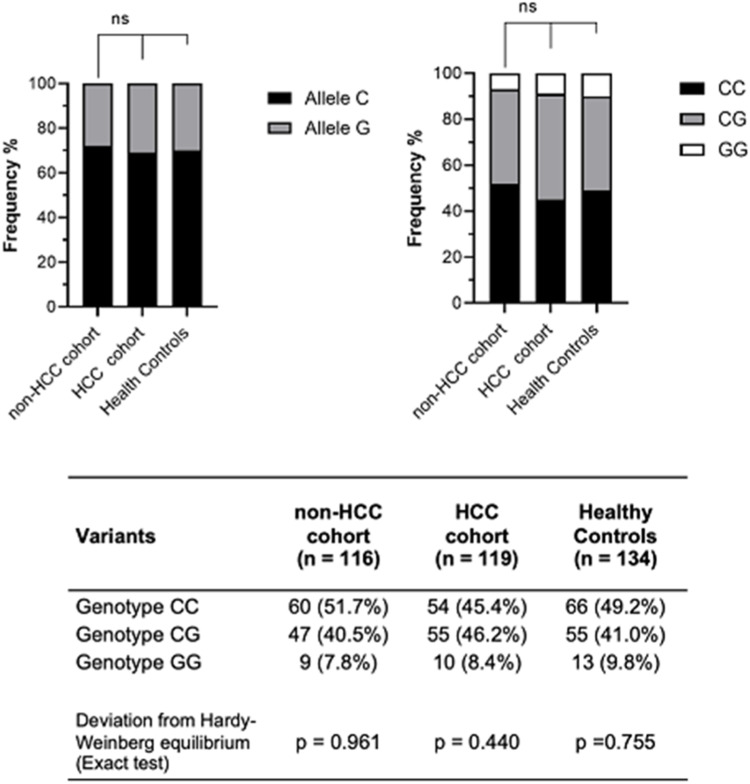
Comparison of the frequencies of *PNPLA3* rs738409 genotypes and Alleles in CHC patients with advanced fibrosis, segmented into HCC and non-HCC cohorts, and healthy controls.

When evaluating the *PNPLA3* polymorphism, the sample was balanced by Hardy-Weiberg equilibrium (Fig. 1). The frequencies of the *PNPLA3* genotypes were CC 45.4 %, CG 46.2 % and GG 8.4 % in the HCC group; CC 51.7 %, CG 40.5 % and GG 7.8 % in the non-HCC group; and CC 49.2 %, CG 41.0 % and GG 9.8 % in the healthy controls. There were no significant differences in the genotype frequencies among the groups by the dominant model (*p* = 0.33) or by the recessive model (*p* = 0.92). When assessing *PNPLA3* genotype frequencies according to HCV genotype, no significant differences were observed between the HCC (*p* = 0.35) and the non-HCC groups (*p* = 0.12).

A sub-analysis was performed comparing patients who achieved SVR before HCC diagnosis with patients who achieved SVR in the non-HCC group (Table 2). The HCC group showed a higher proportion of alcohol dependence (44.9 % vs. 12.2 %, *p* < 0.001), tobacco consumption (67.95 % vs. 21.62 %, *p* < 0.0001), steatosis (53.85 % vs. 28.77 %, *p* = 0.03), and HCV genotype 3 (*p* = 0.021). No statistical differences were observed in the frequencies of *PNPLA3* genotypes by the dominant model (*p* = 0.17) or recessive model (*p* = 0.41).

**Table 2:** Comparison between patients that presented SVR before HCC development vs. non-HCC.

**Variables** (*n* = non-HCC / HCC)		**Groups**			**p**	
		**Non-HCC**	**HCC**			
**Gender n (%)** (*n* = 74 / 79)	Female	45 (60.81 %)	12 (15.19 %)		<0.0001*	
	Male	29 (38.19 %)	67 (84.81 %)			
**Alcohol** (*n* = 74 / 78)	Yes	9 (12.2 %)	35 (44.9 %)		<0.001*	
	No	65 (87.8 %)	43 (55.1 %)			
**Tobacco** (*n* = 74 / 78)	Yes	16 (21.62 %)	53 (67.95 %)		<0.0001*	
	No	58 (78.38 %)	25 (32.05 %)			
**T2DM** (*n* = 74 / 79)	Yes	24 (32.43 %)	21 (26.59 %)		0.43	
	No	50 (67.57 %)	58 (73.41 %)			
**SAH** (*n* = 74 / 79)	Yes	41 (55.40 %)	34 (43.04 %)		0.13	
	No	33 (44.60 %)	45 (56.96 %)			
**Liver steatosis** (*n* = 73 / 65)	Yes	21 (28.77 %)	35 (53.85 %)		0.03*	
	No	52 (71.23 %)	30 (46.15 %)			
**Obesity** (*n* = 72 / 76)	Non-obese	49 (68.06 %)	62 (81.58 %)		0.058	
	Obese	23 (31.94 %)	14 (18.42 %)			
**HCV genotype** (*n* = 74 / 51)	1	56 (76.00 %)		31 (61.00 %)		0.021*
	2	3 (4.00 %)		0		
	3	13 (18.00 %)		20 (39.00 %)		
	4	2 (3.00 %)		0		
**PNPLA3** (*n* = 74 / 79)	CC	40 (54.05 %)		34 (43.04 %)		0.17
	CG/GG	34 (45.95 %)		45 (59.96 %)		

HCC, Hepatocellular Carcinoma; HCV, Hepatitis C Virus; PNPLA3, *Patatin-like Phospholipase domain containing 3*; SAH, Systemic Arterial Hypertension; SD, Standard Deviation; SVR, Sustained Virologic Response; T2DM, Type 2 Diabetes Mellitus.

Assessing the differences within the HCC group according to the presence of the SNP, there were no significant differences in alcohol consumption, liver function at HCC diagnosis, number of nodules, nodules size, degree of tumor differentiation by Edmondson-Steiner, presence of vascular invasion, or presence of steatosis in the non-tumorous liver (Supplementary Table 2).

### Risk factors associated with hepatocellular carcinoma development

Multiple logistic regression analyses assessed which factors were independently associated with HCC development. In the univariate and multivariate analysis, only the variables gender (OR = 4.39, 95 % CI 2.35‒8.39, *p* < 0.001; OR = 3.65, 95 % CI 1.69‒8.17, *p* < 0.001), tobacco (OR = 5.53, 95 % CI 2.91‒10.86, *p* < 0.001; OR = 4.36, 95 % CI 2.16‒9.07, *p* < 0.001) and ALBI (OR = 2.48, 95 % CI 1.35‒4.61, *p* = 0.004; OR = 2.46, 95 % CI 1.23‒5.04, *p* = 0.012) were independent factors associated with the risk of developing HCC (Table 3). The *PNPLA3* G allele was not associated with the risk of HCC in the univariate (*p* = 0.65) or multivariate analysis (*p* = 0.98).

**Table 3:** Univariate and multivariate analysis of factors associated with HCC in CHC patients.

**Variables**		**OR (Univariate)**	**OR (Multivariate)**
Gender	Female	‒	‒
	Male	4.39 (2.35‒8.39, *p* < 0.001)	3.65 (1.69‒8.17, *p* = 0.001)
Alcohol	No	‒	‒
	Yes	3.01 (1.51‒6.32, *p* = 0.002)	1.11 (0.46‒2.70, *p* = 0.817)
Tobacco	No	‒	‒
	Yes	5.53 (2.91‒10.86, *p* < 0.001)	4.36 (2.16‒9.07, *p* < 0.001)
T2DM	No	‒	‒
	Yes	1.03 (0.54‒2.00, *p* = 0.931)	1.14 (0.53‒2.48, *p* = 0.742)
ALBI	1	‒	‒
	2‒3	2.48 (1.35‒4.61, *p* = 0.004)	2.46 (1.23‒5.04, *p* = 0.012)
PNPLA3	CC	‒	‒
	CG/GG	1.15 (0.63‒2.08, *p* = 0.648)	0.99 (0.49‒2.00, *p* = 0.981)

ALBI, Albumin-Bilirubin; HCC, Hepatocellular Carcinoma; HCV, Hepatitis C Virus; OR, Odds Ratio; PNPLA3, *Patatin-like Phospholipase domain containing 3*; T2DM, Type 2 Diabetes Mellitus.

The history of alcohol use disorder was not an independent factor associated with the development of HCC (Table 3). When analyzing without gender and tobacco, a history of alcohol dependence was an independent factor associated with the risk of HCC (OR = 2.96, 95 % CI 1.46‒6.33, *p* = 0.004).

In the sub-analysis of patients who achieved SVR (Table 4), only gender (*p* = 0.001) and tobacco (*p* < 0.001) were independent factors associated with HCC risk. The *PNPLA3* G allele was also not associated with HCC risk in the univariate (*p* = 0.18) or multivariate analysis (*p* = 0.52).

**Table 4:** Univariate and multivariate analysis of factors associated with HCC in CHC patients after SVR.

**Variables**		**OR (Univariate)**	**OR (Multivariate)**
Gender	Female	‒	‒
	Male	7.28 (3.25‒17.37, *p* < 0.001)	4.94 (1.88‒13.95, *p* = 0.002)
Alcohol	No	‒	‒
	Yes	4.22 (1.86‒10.35, *p* = 0.001)	1.21 (0.41‒3.57, *p* = 0.725)
Tobacco	No	‒	‒
	Yes	8.17 (3.70‒19.29, *p* < 0.001)	6.11 (2.48‒16.07, *p* < 0.001)
T2DM	No	‒	‒
	Yes	0.84 (0.39‒1.83, *p* = 0.665)	0.73 (0.27‒1.96, *p* = 0.537)
ALBI	1	‒	‒
	2‒3	2.32 (1.11‒5.02, *p* = 0.028)	2.59 (1.02‒6.98, *p* = 0.050)
PNPLA3	CC	‒	‒
	CG/GG	1.63 (0.81‒3.31, *p* = 0.177)	1.33 (0.55‒3.23, *p* = 0.523)

ALBI, Albumin-Bilirubin; CHC, Chronic Hepatitis C; HCC, Hepatocellular Carcinoma; OR, Odds Ratio; PNPLA3, *Patatin-like Phospholipase domain containing 3*; SAH, Systemic Arterial Hypertension; T2DM, Type 2 Diabetes Mellitus.

A binomial logistic regression model for predicting HCC was conducted, including gender, tobacco consumption, and ALBI. This model achieved an Area Under the Curve (AUC) of 0.74. When the *PNPLA3* polymorphism was included as a variable, there was no improvement in the accuracy for predicting HCC (Supplementary Table 3).

## Discussion

The present study was the first to evaluate the potential association of the *PNPLA3* polymorphism with HCC development in patients with CHC in the Brazilian population. The authors found no difference in the frequencies of the *PNPLA3* genotypes and no significant association between *PNPLA3* and HCC development in this study.

Most previously published studies have been focused on Metabolically Dysfunctional-Associated Liver Disease (MASLD).^16^^,^^17^ Previous data on CHC were quite heterogeneous, with studies that included various degrees of fibrosis and different etiologies, leading to divergent results.^11^^,^^13^^,^^18^ Although some studies have shown positive results, there is no robust data to confirm the association of *PNPLA3* with HCC development in CHC patients.^13^^,^^19^^,^^20^ The published meta-analyses to date were not designed specifically to evaluate CHC; instead, CHC was included as part of the overall analysis or subgroup analysis, often grouped with HBV in viral diseases.^15^^,^^21^^,^^22^

This topic first gained attention in 2011 when Valenti et al. compared 275 CHC patients with various degrees of fibrosis with 50 patients with HCV-related HCC, showing a positive association between the *PNPLA3* GG genotype and HCC development in that cohort.^13^ The same year, a German case-control study, which included only CHC cirrhotic patients, showed a negative association between the *PNPLA3* GG genotype and HCC development.^23^

Subsequently, Singal et al. published a meta-analysis in 2014, in which the association of *PNPLA3* with HCC development in cirrhotic patients was evaluated using both dominant and recessive models. The recessive model presented significant heterogeneity, which differed from the dominant model. In the analysis of all patients, the *PNPLA3* G allele was associated with HCC development. However, when the CHC subgroup was analyzed, no statistically significant association was found.^22^ In the same year, this group published another study that included 937 patients with CHC and varying degrees of fibrosis, showing a negative association between the *PNPLA3* G allele and HCC development. They later updated their previous meta-analysis, including data from this new study, and the association remained negative.^14^ More recently, a large longitudinal study conducted in Taiwan, which included 1011 CHC patients with varying degrees of fibrosis, demonstrated that the *PNPLA3* GG genotype was not associated with the risk of HCC development, regardless of the degree of fibrosis, SVR, or the presence of steatosis.^18^ Focusing solely on cirrhotic patients, Urias et al. demonstrated a significantly higher 5-year cumulative incidence of HCC in those with the GG genotype. However, when stratified by etiology, viral causes did not show a statistically significant association.^24^

In a secondary analysis, the authors evaluated other factors associated with HCC development in addition to the *PNPLA3* polymorphism. The HCC group had significantly more advanced liver disease than the non-HCC group, with higher ALBI scores and more portal hypertension. There is already strong evidence that the degree of liver damage is a significant factor associated with the risk of developing HCC. Masuzaki et al. showed that Liver Stiffness Measures (LSM) can be used to stratify the risk of HCC in CHC patients. Higher LSM was associated with a higher risk, reaching up to 45.5 times higher when LSM > 25 kPa.^25^ In agreement, the Baveno VII Faculty considers that progressive LSM values have an increasing impact on the risk of liver decompensation and liver-related death. In patients with LSM > 25 kPa, it is possible to assume the presence of clinically significant portal hypertension.^26^

Furthermore, the ALBI score was an independent factor associated with HCC development in this study. The ALBI score is an objective tool based exclusively on laboratory parameters that has gained prominence in the assessment of liver function, particularly in patients diagnosed with HCC.^27^ It was developed to overcome the limitations of the Child-Pugh score classification by offering a more refined stratification of patients.^28^ In HCC treatment, it has already been evaluated as a predictor of liver failure and mortality across various therapeutic modalities.^29^ However, its applications now extend beyond the context of HCC treatment.^29^ Importantly, the ALBI score has also demonstrated value in predicting the risk of HCC development in patients with compensated liver cirrhosis, especially those with chronic viral hepatitis.^30^ An Italian retrospective study including 514 patients with CHC cirrhosis who had been treated with DAAs showed that ALBI grades 2 and 3 before HCV treatment presented a three times higher risk of HCC compared to grade 1.^31^ Interestingly, a horizontal Japanese study demonstrated that patients who developed HCC did not show significant differences in ALBI score at baseline compared to other patients. However, at the time of HCC diagnosis, these patients had significantly higher ALBI scores.^32^ This suggests that the ALBI score may help to monitor CHC patients over time, and that increasing values should be considered a warning sign. Thus, the ALBI score may be useful not only for evaluating patients already diagnosed with HCC but also as a surveillance and risk stratification tool in cirrhotic populations.^29^

In addition to evaluating other risk factors, gender and tobacco consumption were also independent factors associated with a higher risk of HCC development, even post-SVR. This association is already well-established in the literature. Men have a higher risk up to 5-fold higher risk than women, depending on the region analyzed.^33^ One of the leading hypotheses for this disparity involves the influence of hormones, where androgens may promote hepatocarcinogenesis by activating pathways that enhance hepatic inflammation and cellular proliferation, while estrogens appear to exert a protective effect by inhibiting angiogenesis, fibrogenesis, and inflammatory responses.^34^ Additionally, immunological differences contribute to this disparity, as women tend to mount stronger innate and adaptive immune responses.^35^ Behavioral factors also play an important role because men have higher exposure rates to hepatocarcinogenic agents such as alcohol, Tobacco, and aflatoxins.^33^ As for tobacco, it can induce carcinogenic mutations, contributing as an additional risk factor for HCC development.^36^ Mori et al. demonstrated that there is a significant additive interaction between tobacco use and CHC in the risk of developing HCC.^37^ A meta-analysis showed that non-smoking patients with HCV had a relative risk of 7.94 for developing HCC. However, when HCV infection was combined with smoking, the relative risk increased to 23.1, confirming the previously described additive effect on HCC risk.^38^

Interestingly, in the present study, alcohol dependence was not an independent factor associated with the development of HCC when analyzed alongside the variables sex and tobacco. This was due to multicollinearity, which prevented the model from distinguishing the individual effect of each factor. In previously published studies, patients with Chronic Hepatitis C (CHC) and alcohol consumption presented a 2-fold risk of developing HCC, which may occur at an earlier age and with potentially more advanced tumors.^39^

T2DM showed no significant difference between the groups and was not an independent factor associated with HCC risk in the present cohort. Most studies that have demonstrated a synergic role of T2DM in HCC risk have compared patients with HCC to the general population.^37^^,^^40^ Other case-control studies focusing exclusively on patients with advanced fibrosis, such as ours, also did not observe this association.^19^^,^^23^ This suggests that T2DM may contribute to the progression of liver disease in CHC patients, thereby increasing the risk of HCC development.

As this is a cross-sectional, single-center study with retrospective analysis, it is important to highlight some limitations. The authors acknowledge that this design and the sample size are not ideal for assessing the impact of SNPs in a population. A longitudinal prospective study is required to clearly demonstrate the negative association between the PNPLA3 polymorphism and HCC development. Furthermore, as a retrospective study, there were missing data in both cohorts. Nevertheless, this study is the first to assess the impact of the *PNPLA3* rs738409 *C* > *G* polymorphism on HCC development in CHC patients in the Brazilian population. Moreover, the authors only included patients with advanced fibrosis, reducing the risk of bias due to sample heterogeneity, and the mean follow-up time for the non-HCC patients in the outpatient units was approximately 7-years.

## Conclusion

The *PNPLA3* rs738409 *C* > *G* polymorphism does not seem to be associated with the risk of HCC development in CHC patients in the Brazilian population. Further studies with a prospective design are required to confirm these findings. Additional risk factors should also be evaluated to enhance risk stratification for HCC development in CHC patients. Meanwhile, other risk factors such as the ALBI score and tobacco consumption may play an important role in the HCC risk stratification of CHC patients.

## Authors’ contributions

CM: Data collection, concept and design, data interpretation and drafting of the article; IVP: Concept and design, statistical analysis, data interpretation and critical revision; LRCS: Data collection; RSSMA: Data collection; AINO: Data collection; JTS: Concept and design, critical revision; MSGG: Experiments and procedures; JMKSE: Experiments and procedures; PH: Sample collection and critical revision; JRRP: Experiments and procedures; RSNP: Sample collection and critical revision; WA: Sample collection and critical revision; LACA: Sample collection and critical revision; MGP: Concept and design and critical revision; ALP: Concept and design, data interpretation and critical revision; CPMSO: Concept and design, critical revision and final approval.

## Funding

This study was financed in part by the Coordenação de Aperfeiçoamento de Pessoal de Nível Superior Brasil (CAPES) – Finance Code 001

## Declaration of competing interest

The authors declare no conflicts of interest.

## Appendix Supplementary materials

Image, application 1
