# Supplementary material for: The impact of the PNPLA3 rs738406 C>G polymorphism on hepatocellular carcinoma risk in Brazilian patients with chronic hepatitis C and advanced fibrosis
Source: Clinics (Sao Paulo). 2025 Oct 14;80:100797. doi: 10.1016/j.clinsp.2025.100797 (PMC12550332; doi:10.1016/j.clinsp.2025.100797)
Supplement: Supplementary file 1 [file mmc1.docx]

**CLINICS-D-25-00595_Supplementary Material**

**Supplementary Table 1** Cirrhotic patients’ clinical characteristics.

| **Variables**  (n = non-HCC/HCC) | | **Groups** | | **p** |
| --- | --- | --- | --- | --- |
|  |  | **Non-HCC** | **HCC** |  |
| **Child-Pugh**  (n = 85/ 119) | A | 77 (90.59%) | 97 (81.51%) | 0.07 |
|  | B/C | 8 (9.41%) | 22 (18.49%) |  |
| **MELD**  (n = 78/ 113) | < 15 | 73 (93.59) | 101 (89.38%) | 0.44 |
|  | ≥ 15 | 5 (6.41%) | 12 (10.62%) |  |
| **ALBI**  (n = 76/ 111) | 1 | 50 (65.79%) | 47 (42.34%) | 0.002* |
|  | 2‒3 | 26 (34.21%) | 64 (57.66%) |  |
| **Portal hypertension**  (n = 89/ 119) | Yes | 58 (65.17%) | 92 (77.30%) | 0.05 |
|  | No | 31 (34.83%) | 27 (22.70%) |  |

ALBI, Albumin-Bilirubin; HCC, Hepatocellular Carcinoma; MELD, Model for End-stage Liver Disease; SD, Standard Deviation.

**Supplementary Table 2** Clinical characteristics of HCC cohort by PNPLA3 genotype.

| **Variables**  (n = CC / CG/GG) | | **PNPLA3** | | **p** |
| --- | --- | --- | --- | --- |
|  |  | **CC** | **CG/GG** |  |
| **Alcohol consumption >140 g/w**  (n = 54 / 62) | Yes | 20 (37.04%) | 37 (59.98%) | 0.72 |
|  | No | 34 (62.96%) | 25 (40.32%) |  |
| **Child-Pugh**  (n = 54 / 65) | A | 43 (79.63%) | 54 (83.08%) | 0.73 |
|  | B/C | 11 (20.37%) | 11 (16.92%) |  |
| **MELD**  (n = 51 / 62) | ≤ 15 | 48 (94.12%) | 57 (91.93%) | 0.27 |
|  | > 15 | 3 (5.88%) | 5 (8.07%) |  |
| **ALBI**  (n = 50 / 61) | 1 | 21 (42.00%) | 26 (42.63%) | 0.29 |
|  | 2‒3 | 29 (58.00%) | 35 (57.37%) |  |
| **Portal hypertension**  (n = 54 / 65) | Yes | 43 (79.63%) | 49 (75.39%) | 0.58 |
|  | No | 11 (20.37%) | 16 (24.61%) |  |
| **Nodules at diagnosis**  (n = 54 / 65) | 1 | 40 (74.07%) | 49 (75.39%) | 0.65 |
|  | 2‒3 | 11 (20.37%) | 12 (18.46%) |  |
|  | ≥ 4 (multifocal) | 3 (5.56%) | 4 (6.15%) |  |
| **Major nodule size (cm)**  (n = 54 / 65) | Mean + SD | 30.89 ± 16.88 | 30.37 ± 19.15 | 0.88 |
| **Sum of all nodules** **(cm)**  (n = 54 / 65) | Mean + SD | 40.37 ± 28.83 | 38.11 ± 24.98 | 0.65 |
| **BCLC at diagnosis**  (n = 54 / 65) | 0 | 13 (24.07%) | 11 (16.92%) | 0.14 |
|  | A | 29 (53.70%) | 47 (72.30%) |  |
|  | B | 8 (14.82%) | 5 (7.70%) |  |
|  | C/D | 4 (7.41%) | 2 (3.08%) |  |
| **Milan criteria at diagnosis**  (n = 54 / 65) | Yes | 43 (79.63%) | 57 (87.69%) | 0.23 |
|  | No | 11 (20.27%) | 8 (12.31%) |  |
| **Degree of tumoral differentiation**  (n = 39 / 51) | I/II | 18 (46.15%) | 21 (41.18%) | 0.64 |
|  | III/IV | 21 (53.85%) | 30 (58.82%) |  |
| **Vascular invasión**  (n = 42 / 55) | Yes | 14 (33.33%) | 25 (45.45%) | 0.23 |
|  | No | 28 (66.67%) | 30 (54.55%) |  |
| **Liver steatosis**  (n = 46 / 53) | Yes | 19 (41.30%) | 29 (54.71%) | 0.18 |
|  | No | 27 (58.70%) | 24 (45.29%) |  |

ALBI, Albumin-Bilirubin; BCLC, Barcelona Clinic Liver Cancer; CM, Centimeters; HCC, Hepatocellular Carcinoma; MELD, Model of End stage Liver Disease; PNPLA3, *Patatin-like Phospholipase domain containing 3*; SD, Standard Deviation.

**Supplementary Table 3** Binominal logistic regression model for HCC prediction in CHC patients.

|  | | | | | | **95% CI** | |
| --- | --- | --- | --- | --- | --- | --- | --- |
| **Predictor** | **Estimate** | **SE** | **Z** | **p** | **OR** | **Inferior** | **Superior** |
| **Intercept** | -1.966 | 0.41 | 4.777 | <0.001 | 0.140 | 0.062 | 0.314 |
| **Gender:** |  |  |  |  |  |  |  |
| Male – Female | 1.360 | 0.36 | 3.736 | <0.001 | 3.897 | 1.909 | 7.956 |
| **Tobacco** |  |  |  |  |  |  |  |
| Yes – No | 1.709 | 0.36 | 4.754 | <0.001 | 5.526 | 2.731 | 1.118 |
| **ALBI:** |  |  |  |  |  |  |  |
| 2‒3 – 1 | 0.816 | 0.36 | 2.269 | 0.023 | 2.262 | 1.118 | 4.579 |
| **PNPLA3:** |  |  |  |  |  |  |  |
| GG, CG – CC | 0.215 | 0.36 | 0.597 | 0.55 | 1.239 | 0.613 | 2.506 |

ALBI, Albumin-Bilirubin; CHC, Chronic Hepatitis C; CI, Confidence Interval; HCC, Hepatocellular Carcinoma; OR, Odds Ratio; PNPLA3, *Patatin-like Phospholipase Domain Containing 3*; SE, Standard Error.
